# The Role of Dendritic Cells in the Differentiation of T Follicular Helper Cells

**DOI:** 10.1155/2018/7281453

**Published:** 2018-07-02

**Authors:** Xueyang Zou, Guangyu Sun, Feifei Huo, Lu Chang, Wei Yang

**Affiliations:** Department of Immunology, College of Basic Medical Sciences, Jilin University, Changchun 130021, China

## Abstract

T follicular helper cells (T_FH_) are a subset of recently discovered CD4^+^ T cells. Their major function is to participate in the formation of germinal centres (GCs) and promote B cell proliferation and differentiation to play important roles in the production of antibodies. Currently, the functions of T_FH_ cells are clear. However, the early differentiation of these cells is not clear. Dendritic cells (DCs) participate in the differentiation of T_FH_ cells. Therefore, this article reviewed the research progress regarding the influence of DCs on the differentiation of T_FH_ cells and their major underlying mechanisms.

## 1. Introduction

In the early 20th century, people first discovered a group of special T cells in the tonsils. These cells were localised in lymphoid follicles and had the function of assisting in B cell differentiation and maturation. Based on their localisation and function, this group of T cells was named T follicular helper (T_FH_) cells. The characteristic transcription factor of T_FH_ cells is B cell lymphoma 6 (Bcl6). In addition, these cells also express inducible costimulator (ICOS) and C-X-C chemokine receptor type 5 (CXCR5); therefore, they can migrate to B cell follicles to continuously differentiate into germinal centre (GC) T_FH_ [1]. T_FH_ cells primarily exert their function through the secretion of interleukin 21 (IL-21). IL-21 binds to the corresponding receptor on the B cell surface to assist B cells in producing high-affinity antibodies. In addition, IL-21 can also enhance the Bcl6 expression in T cells in an autocrine fashion to promote T_FH_ development.

The process of T_FH_ cell differentiation involves many factors. Naïve T cells interact with dendritic cells (DCs) and differentiate into T_FH_ cells under the functions of three types of signals. The first signal is the interaction between the antigenic peptides presented by the DC/MHCII molecule complex and T cell receptors (TCRs). The second signal is the interaction between the costimulatory molecules on the surface of DCs and the corresponding ligands on the surface of T cells. Currently, the major interactions that have been discovered include the inducible costimulator ligand- (ICOSL-) ICOS and OX40 ligand- (OX40L-) OX40. The third signal is the cytokines secreted by DCs. These known cytokines include mouse IL-6 and IL-27 as well as human IL-12, IL-23, and transforming growth factor-*β* (TGF-*β*). Therefore, this article reviewed the research progress made concerning the promotion of the differentiation of naïve T cells into T_FH_ cells by DCs with regard to these three aspects.

## 2. DCs Affect the T_FH_ Differentiation during Antigen Presentation

The key of initiation of any CD4^+^ T cell response is the recognition of the antigenic peptide/MHCII molecule complex on antigen-presenting cells (APCs) by TCR. DCs are currently recognised as the most powerful APCs in the body. The sizes of antigens presented by DCs can control their interaction with T cells to drive T_FH_ cell development [2].

When the antigen is larger, it is easier for DCs to uptake the antigen. Benson et al. used an adoptive transfer model to cross hCD2-DsRed (hCD2-red fluorescent protein) mice with ovalbumin- (OVA-) specific OT-II TCR transgenic (Tg) mice. Through a tail vein injection, 2-3 × 10^6^ B cells and 2-3 × 10^6^ T cells were adoptively transferred to age-matched C57BL/6J recipient mice. Next, 40 nm or 200 nm particle-coupled OVA-HEL conjugates were used to stimulate these mice. The results showed that after five days of immunisation by 40 nm particle-conjugated antigens, the percentage of antigen-specific T cells that differentiated into T_FH_ cells was lower. However, the percentage of T_FH_ cells significantly increased after CD4^+^ T cells were stimulated by 200 nm particle-conjugated OVA-HEL. The results suggest that because large-molecule antigens were conducive to DC uptake, they were processed by DCs to present them to T cells to promote T_FH_ cell differentiation [2].

Furthermore, larger antigen particles cannot change the presenting ability of DCs but do prolong the contact time between the antigenic peptides presented by DCs and MHCII molecules [2]. Benson et al. covalently linked a traceable antigen, E*α*GFP, to such particles [2]. Next, the 40 nm or 200 nm particles covalently linked with antigens were subcutaneously injected into C57BL/6 mice. The DCs rapidly presented both antigens in mice after 6 h of both types of immunisation. E*α*/MHCII-positive DCs corresponding to the 40 nm particle antigen formulations were detected at 24 and 48 h. Compared with the 40 nm particle preparations, the duration of antigen presentation by DCs after immunisation with 200 nm particles was over 48 h, and 21 ± 3% of the DCs continued to present antigens after 72 h of immunisation. That study showed that a longer time of contact between peptides and MHCII molecules was conducive to the interaction between late-stage T cells and DCs [2]. Further study suggested that large-molecule antigens also prolong the interaction time between T cells and DCs to further influence T_FH_ differentiation [2].

In summary, the increase in particle sizes and the maintenance of the presenting function of DCs and their interaction with T cells are key factors for promoting T_FH_ differentiation. Particles might influence the process of antigen uptake to affect endogenous targeting, processing, and the expression of antigens. Both the enhancement of the usability of TCR ligands and the TCR signal strength variation can promote T_FH_ differentiation. However, how particle size influences the contact time between antigenic peptides on DC/MHCII molecules remains unclear.

## 3. Costimulatory Molecules Expressed on DCs Regulate T_FH_ Differentiation

**Figure 1:**
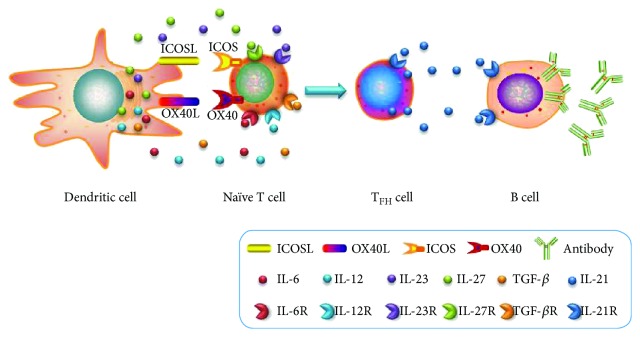
The regulation of T_FH_ differentiation by DCs via the costimulatory and cytokine signal pathway. The costimulatory molecules expressed on the surface of DCs, such as ICOSL and OX40L, and cytokines (IL-6, IL-12, IL-23, IL-27, and TGF-*β*) secreted by DCs can promote the differentiation of naïve T cells into T_FH_ cells. B cells were stimulated to produce antibodies with the help of T_FH_ secreting IL-21. In addition, IL-21 produced by T_FH_ could also enhance T_FH_ differentiation at an autocrine manner.

T_FH_ differentiation requires not only the antigens presented by DCs but also the costimulatory molecules to provide the second signal for the complete differentiation of T_FH_. The current research shows that the major costimulatory molecules on DCs for the induction of T_FH_ differentiation are ICOSL and OX40L (Figure 1).

### 3.1. ICOSL

ICOSL (B7RP-1) is primarily expressed in DCs, B cells, and other APCs. It is a B7 family member. Its ligand, ICOS, is only expressed in activated T cells, especially GC T_FH_ cells, and it belongs to the CD28 family. The ICOSL-ICOS interaction regulates humoral immunity and GC responses. Akiba et al. used sheep red blood cells (SRBCs) to immunise anti-ICOSL monoclonal antibody- (mAb-) treated or ICOS-deficient mice. The results showed that the development of T_FH_ cells in the spleen was significantly impaired. In addition, the T_FH_ cell development in mouse lymph nodes treated with ICOSL mAb was also suppressed. These results indicate that the ICOSL-ICOS interaction plays an important role in the T_FH_ cell development of mice [3]. Interestingly, compared with normal individuals, the percentage of circulating T_FH_ cells in ICOS-deficient patients is also severely decreased [4]. During DC-T cell interaction, the use of an ICOSL-blocking mAb to block ICOSL in DCs reduced the expressions of CXCR5 and ICOS in CD4^+^ T cells [5], suggesting that the ICOSL in DCs participates in T_FH_ cell differentiation and development through an interaction with ICOS in CD4^+^ T cells.

The ICOSL-ICOS interaction is necessary for IL-21 expression in T_FH_ cells. Together, ICOS, TCR, and CD28 promote the expression of nuclear factor of activated T cells (NFAT) through the phosphoinositide 3-kinase (PI3k) pathway. NFAT binds to the IL-21 promoter to regulate IL-21 expression [6]. Moreover, ICOS deficiency significantly inhibits Bcl6 induction to further inhibit CXCR5 expression, indicating that the ICOSL-ICOS signalling pathway guides Bcl6 expression in T cells to promote CXCR5 expression in CD4^+^ T cells and eventually induce T_FH_ differentiation during the initiation of T cell differentiation by DCs [7]. Therefore, the interaction between the ICOSL in DCs and the ICOS in T cells regulates T_FH_ cell differentiation by influencing IL-21 production and Bcl6 expression.

The NF-*κ*B pathway primarily affects the ICOSL expression in DCs. A chromatin immunoprecipitation (ChIP) experiment showed that the p52 in the noncanonical NF-*κ*B pathway significantly enriches the upstream promoter region of the ICOSL in DCs. In addition, application of the NIK inhibitor significantly decreases the ICOSL expression in DCs. These results indicate that DCs enhance ICOSL expression through the noncanonical NF-*κ*B pathway to further mediate DCs to influence T_FH_ cell differentiation [5].

### 3.2. OX40L

OX40L (CD252) and OX40 (CD134) belong to the tumour necrosis factor (TNF) superfamily and the TNF receptor (TNFR) superfamily, respectively. Flow cytometry and immunofluorescence microscopy have shown that OX40L is expressed in the marginal zone bridging channels (MZBCs) of the spleen and the DCs at the edge of B cell follicles. Its receptor OX40 is expressed in both the effector CD4^+^ T cells that will become T_FH_ cells and mature T_FH_ cells. Many years ago, in vitro experiments showed that CXCR5 was not expressed in naïve CD4^+^ T cells; however, after three days of costimulation with OX40L in nonparental cell lines, the mRNA of the T_FH_ cell marker CXCR5 was strongly upregulated [8]. Studies of OX40-deficient mice have shown that on day 8 of acute vaccinia virus (VacV) infection, a large amount of CD44^hi^CD62L^lo^CXCR5^+^ PD-1^+^ or CD44^hi^CD62L^lo^CXCR5^+^ Bcl6^+^ T_FH_ cells was detected in wild-type (WT) mice. However, the number of T_FH_ cells in OX40-deficient mice was reduced by 70% compared with that in the WT mice, suggesting that OX40 and OX40L help T_FH_ development [9]. Recently, Boettler et al. showed that T_FH_ cells decreased, and GC B cell responses, antibody responses, and CD4^+^ and CD8^+^ T cell responses were completely damaged in OX40-deficient mice during persistent lymphocytic choriomeningitis virus (LCMV) infection [10]. In vitro studies have shown that, during the stimulation of T cells from systemic lupus erythematosus patients using anti-CD3/CD28 antibodies, the application of soluble OX40L increases the expressions of T_FH_-related molecules in T cells, including Bcl6, CXCR5, and IL-21, to promote their differentiation into T_FH_ cells [11]. These data also indicated that the OX40-OX40L interaction influences T_FH_ cell differentiation. During the DC-T cell interaction, blocking OX40L in CD8*α*^−^ DCs using the OX40L-blocking mAb reduces the CXCR5 and ICOS expressions in CD4^+^ T cells [5], which suggests that the OX40L in DCs participates in T_FH_ cell differentiation and development through an interaction with OX40 in CD4^+^ T cells.

Studies have shown that OX40 is a strong activator of the PI3K and Akt signalling pathways that depend on the adaptor protein TNF receptor-associated factor 2 (TRAF2) and the NF-*κ*B signalling pathway in CD4^+^ T cells. As a downstream serine/threonine kinase of ICOS, TANK-binding kinase 1 (TBK1) participates in GC T_FH_ development; in addition, it is associated with TRAF2. Therefore, it plays a role in the NF-*κ*B response and acts as an upstream molecule of Akt under certain conditions. Thus, although TBK1 has not been found downstream of OX40, OX40 might recruit TBK1 when the OX40L in DCs interacts with OX40 in CD4^+^ T cells, and the OX40 signal transduction could target ICOS downstream intercellular pathways, which might explain its collaboration with ICOS in the regulation of late-stage T_FH_ responses. In addition, OX40 also assists T_FH_ cells through NFAT. The losses of NFAT1 and NFAT2 in CD4^+^ T cells impair the expression of T_FH_ differentiation-associated molecules such as ICOS and PD-1 [12]. OX40 enhances the nuclear accumulation of NFAT in the CD4^+^ T cells driven by antigens [9]. NFAT1 and NFAT2 acquire calcium signalling by binding to TCR to cause phosphorylation; then, they bind to proximal promoters, binding sites in the transcription regions of the ICOS and IL-6R*α* genes, or both to promote ICOS and IL-6 expression, thereby participating in effector T_FH_ cell development [12].

Shin et al. showed that the p52 in the noncanonical NF-*κ*B pathway significantly enriches the upstream promoter region of OX40L in DCs. After the application of the NIK inhibitor, OX40L expression was significantly decreased, which suggests that DCs enhance OX40L expression through the noncanonical NF-*κ*B pathway [5].

## 4. The Cytokines Secreted by DCs Regulate T_FH_ Differentiation

In addition to the antigenic peptides presented by the DC/MHCII molecule complex and the costimulatory molecules on the surface of DCs, the cytokines secreted by DCs are also important for the early development of T_FH_ cells. Recent studies have shown that the major T_FH_ cell development-associated cytokines in mice and humans are not completely identical. In mice, the IL-6 and IL-27 secreted by DCs play a leading role, and IL-12 also participates in the early stage of T_FH_ differentiation. By contrast, human T_FH_ differentiation is primarily regulated by three cytokines, including IL-12, IL-23, and TGF-*β* (Figure 1).

### 4.1. IL-6

The IL-6 produced by DCs is important for the early differentiation of T_FH_. During the initiation of the CD4^+^ T cell responses by DCs, CD4^+^ T cells promote T_FH_ gene expression in the presence of IL-6; in other words, the expressions of Bcl6 and CXCR5 mRNAs are both upregulated. Chakarov and Fazilleau [13] and Choi et al. [14] showed that the loss of IL-6 in DCs results in T_FH_ cell differentiation defects, which suggests that DCs secrete IL-6 to participate in T_FH_ cell development.

IL-6 induces Bcl6 expression and mediates T_FH_ differentiation through signal transducer and activator of transcription (STAT3) [14]. STAT3 is the first activated transcription factor downstream of the IL-6 receptor. WT and STAT3^−/−^ SM CD45.1^+^ SMARTA (“SM” LCMV gp66–77-IAb-specific) cells were transferred into B6 mice. After two days of LCMV infection, the induction of Bcl6 in STAT3^−/−^ SM cells showed defects, and the number of T_FH_ cells decreased [15]. The above results suggest that STAT3 participates in the induction of Bcl6 expression downstream of IL-6R signalling to mediate T_FH_ differentiation.

STAT1 is an important transcription factor to promote IL-6-dependent Bcl6 induction and T_FH_ differentiation during the DC priming stage of acute viral infections [14]. When the IL-6 secreted by DCs binds to IL-6R in CD4^+^ T cells to activate the STAT3 in various types of haematopoietic cells, the STAT1 in CD4^+^ T cells is also selectively activated by IL-6. When the STAT1 expression is inhibited by a specific shRNA miR (i.e., STAT1^KD^ hereafter), STAT1^KD^ SM cells cannot be differentiated into T_FH_ cells after two days of vaccinia virus- (VACV-) gpc infection. Overall, the above data indicate that STAT1 is an important transcription factor that guides IL-6-dependent Bcl6 expression and T_FH_ differentiation during the DC priming stage of acute viral infections [14].

### 4.2. IL-12

IL-6 and IL-21 are important for T_FH_ cell formation in mice. In humans, the IL-12 produced by activated DCs is required for the induction of the differentiation of naïve CD4^+^ T cells into T_FH_ cells [16]. IL-12R*β*1 is the IL-12 receptor; under its defect, the circulating memory T_FH_ cells and memory B cells are significantly reduced. In vitro studies of human cells have suggested that the ability of IL-12 to induce human naïve CD4^+^ T cells to express IL-21 is stronger than those of IL-6 and IL-21. The following points together explain the importance of IL-12 in the human body regarding the development of T_FH_ cells that produce IL-21: (1) the soluble factors secreted by activated DCs effectively induce naïve CD4^+^ T cells to become cells that secrete IL-21; (2) IL-12 is the most effective cytokine from DCs that induces IL-21 production in CD4^+^ T cells; (3) TLR ligand, CD40L, and bacteria-activated DCs secrete IL-12 and effectively induce naïve CD4^+^ T cells to secrete IL-21; (4) blocking IL-21 during the coculture of activated DCs and naïve CD4^+^ T cells results in a significant reduction of the IL-21 secretion by CD4^+^ T cells; and (5) in vitro CD4^+^ T cells produced after naïve CD4^+^ T cells cultured with IL-21 can assist B cells to secrete antibodies in an IL-21-dependent manner. In addition, IL-12 is a critical factor not only for developing CD4^+^ T cells that produce IL-21 but also for helping memory CD4^+^ T cells to secrete IL-21. Therefore, human DCs likely regulate T_FH_ differentiation and development through the secretion of IL-12 [17].

The regulation of T_FH_ development via IL-12 largely depends on STAT4. Nakayamada et al. showed that expression of Bcl6 induced by IL-12 was significantly suppressed in Stat4-deficient (Stat4^−/−^) T cells, which suggests that STAT4 mediates IL-12-dependent Bcl6 expression. Studies have also shown that the percentage of CXCR5^+^PD-1^+^cells and expression of ICOS were also significantly reduced in Stat4^−/−^ T cells [18]. Qiu et al. suggested that IL-12-mediated STAT4 signalling participates in the production of important transcription factors in T_FH_ cells such as Bcl6, c-Maf, and BATF [19]. In human cells, however, IL-12 does not seem to show the same function as it does in mouse cells. Many studies have found that IL-12 does not induce mouse naïve CD4^+^ T cells to secrete IL-21 [20].

### 4.3. IL-23

IL-23 is a proinflammatory cytokine. It is a member of the IL-12 molecule family, together with IL-21 and IL-27. IL-23 is produced by DCs, and it is important for the early differentiation of human T_FH_ cells. Current studies have shown that the signal transduction mediated by the IL-12 and IL-23 common receptor, IL-12 receptor *β*1 chain (IL-12R*β*1), is important for the T_FH_ responses in the human body [21]. Compared with a control group, people with IL-12R*β*1 deficiency have fewer circulating memory T_FH_ cells and memory B cells in the peripheral blood as well as showing GC formation impairment in their lymph nodes.

IL-23 promotes IL-21 and CXCR5 expressions in T_FH_ cells. NanoString analyses of transcription factor expression have shown that IL-23 effectively induces the expression of Bcl6 transcripts on T_FH_ cells. Analyses at the protein level using flow cytometry further confirmed that IL-23 induces the expression of Bcl6 rather than of Blimp-1 (encoded by PRDM1) on T_FH_ cells [21]. IL-23 regulates Bcl6 expression through the JAK-STAT signalling pathway [22]. The interaction between the IL-23 secreted by DCs and the corresponding receptor IL-12R*β*1 in CD4^+^ T cells induces receptor molecule dimerisation to approximate the receptor-coupled JAK kinase and cause activation through interactive tyrosine phosphorylation. JAK activation catalyses the phosphorylation modification of the tyrosine residuals on the receptor. These phosphorylated tyrosine sites and the surrounding amino acid sequence form a “docking site.” In addition, STAT3 proteins containing the SH3 domain are recruited to this “docking site.” Finally, JAK kinase catalyses the phosphorylation modification of receptor-binding STAT3 proteins. The activated STAT3 protein enters a cell nucleus as a dimer to bind to the Bcl6 gene to regulate Bcl6 transcription and further regulate T_FH_ differentiation. One study suggested that IL-23 also promotes the expression of the transcription factor Batf, which is important for T_FH_ cell production; however, the specific mechanisms await further study [23].

### 4.4. IL-27

IL-27 is an IL-12 family member that is composed of Epstein-Barr virus-induced gene3 (EBI3) and the p28 subunit. The former is shared with IL-12 p35, and the latter is the specific subunit of IL-27. IL-27 is primarily produced by DCs, monocytes, and macrophages. Compared with the T cells in WT mice, the CD4^+^ T cells of OVA/CFA-immunised IL-27Ra^−/−^ mice secreted less IL-21. One study showed that IL-27Ra^−/−^ mice had an inherent defect of T_FH_ differentiation, which further indicates the important functions of IL-27 in T_FH_ differentiation [24].

Studies have shown that the IL-27 in DCs stimulated by anti-CD3/CD28 antibodies binds to its receptor (which is composed of two chains, including the gp130 shared with IL-6 and the IL-27Ra-specific chain) to activate Janus kinase (JAK/STAT3) and mitogen-activated protein kinase (MAPK) signals as well as increase the IL-21 production in naïve T cells to further promote T_FH_ differentiation [25]. Other studies have also suggested that the IL-27 produced by the DCs driven by fucose-specific DC-SIGN induces T_FH_ cell polarisation [26]. Through IKK*ε*- (I*κ*B kinase) dependent interferon-stimulated gene factor 3 (ISGF3), DC-SIGN signals activate and recruit RNAP2 to accelerate IL-27 transcription and specifically upregulate IL-27p28. To further elucidate the function of DC-SIGN-induced IL-27 on T_FH_ polarisation after DC priming, Gringhuis et al. used antibodies to neutralise IL-27 during the coculture of DC-T cells. The results showed that the DCs stimulated by LPS and fucose together did not induce T_FH_ polarisation. In addition, similar to neutralising IL-27 antibodies, blocking DC-SIGN-mediated ISGF3 activation also inhibited Bcl6 induction and IL-21 secretion in T_FH_ cells. Therefore, these results also indicate that the DC-SIGN-induced IL-27 expression in DCs is important for the induction of T_FH_ polarisation [26].

### 4.5. TGF-*β*

TGF-*β* is another cofactor that has been confirmed as important for the early differentiation of human T_FH_ cells. TGF-*β* is produced and secreted by DCs; together with IL-12 and IL-23, it promotes the expression of many T_FH_ molecules (including CXCR5, ICOS, IL-21, Bcl6, BATF, and c-Maf) and downregulates the expression of the transcription factor BLIMP-1, which mutually antagonises with Bcl6. However, whether TGF-*β* promotes T_FH_ development in mice has not been verified.

Current studies have indicated that IL-12 and IL-23 both help the production of human T_FH_ cells. TGF-*β* is a key cofactor of IL-12 and IL-23 to promote T_FH_ cell differentiation in humans. When TGF-*β* is combined with IL-12 and IL-23, the T_FH_ transcription signature (i.e., the upregulation of Bcl6, c-Maf, and BATF as well as the downregulation of Blimp-1) is strongly induced [27].

STAT3 and STAT4 participate in the regulation of T_FH_ cell differentiation via TGF-*β* [27]. In human helper T cells, IL-12 and IL-23 separately deliver major activation signals through STAT4 and STAT3, respectively [28]. TGF-*β* can use two methods to collaborate with STAT3 and STAT4 in the initial differentiation of human T_FH_ cells [27]. First, although TGF-*β* itself lacks the ability to induce T_FH_, it can enhance the ability of STAT3-STAT4 to induce human naïve CD4^+^ T cells to express T_FH_ cell characteristic molecules including CXCR5, ICOS, IL-21, Bcl6, Batf-Jun, and c-Maf. More importantly, this stimulation function of TGF-*β* on T_FH_ development seems to be confined to human CD4^+^ T cells and does not have effects on mouse CD4^+^ T cells. Studies using mouse models have indicated that the blockage of TGF-*β* in DCs does not reduce CXCR5 expression in activated CD4^+^ T cells or GC B cell formation [13]. Second, TGF-*β* inhibits human naïve CD4^+^ T cells from expressing Blimp-1. In the presence of TGF-*β*, Blimp-1 expression is further downregulated through STAT3-STAT4 to convert the balanced state between Bcl6 and Blimp-1 into a state of Bcl6 dominance to further promote T_FH_ differentiation. Many studies have indicated that TGF-*β* inhibits the expressions of Bcl-6 [29] (miR-10a inhibits Bcl-6 expression, whereas TGF-*β* promotes miR-10a expression to further inhibit Bcl-6 expression [30]) and IL-21 [31] in mouse CD4^+^ T cells. These observational results suggest that the TGF-*β* secreted by DCs in mice and humans has different effects on T_FH_. The positive regulation of TGF-*β* on T_FH_ formation might be only limited to human T cells, whereas TGF-*β* has shown inhibitory effects on the formation and function of mouse T_FH_ cells.

## 5. SAP Is Involved in T_FH_ Differentiation

In addition to the factors mentioned above, another signalling molecule, SLAM-associated protein (SAP), is important to allow mouse T_FH_ differentiation. SAP-deficient (Sh2d1a^−/−^) T_FH_ cells appeared to resemble the wild type yet failed to induce GCs in vivo [32]. Cannons et al. found that T_FH_ cell markers, PD-1 and CXCR5, were upregulated in the Sh2d1a^−/−^T cells during the initial response (day 4). However, when GC formation was maximal (days 7–9), the percentage of T_FH_ was significantly reduced in Sh2d1a^−/−^ mice. When Sh2d1a^−/−^OT-II GFP^+^ T cells were transferred into the Sh2d1a^−/−^ host, the number of T_FH_ cells was also decreased after NP-OVA immunisation [33]. Studies showed that SAP-deficient T_FH_ cells appeared virtually indistinguishable from the wild type early in the response, but the number of T_FH_ cells was significantly different at the time of maximal GC formation [33].

To assess the ability of Sh2d1a^−/−^ T_FH_ to initiate and sustain GC responses in vivo, Lu et al. adoptively transferred low numbers (4 × 10^4^) of GFP^+^ OT-II T cells into Sh2d1a^−/−^ recipients lacking GCs. The results showed that GC formation can be rescued in Sh2d1a^−/−^ recipients receiving T_FH_ cells from WT mice, but not from Sh2d1a^−/−^ mice [32].

SAP-deficient CD4^+^ T cells are activated normally by DCs and initially express T_FH_ markers. Using intravital imaging, the researchers demonstrated that Sh2d1a^−/−^ CD4^+^ T cells interact with and are activated effectively by antigen-bearing DCs, resulting in proliferation and upregulation of activation markers and migration into B cell follicles, similar to WT CD4 T cells [33]. The mechanism of this phenomenon and the ligands in DCs responsible for interacting with SAP are still unknown. In addition, whether DCs can affect the expression of SAP to T_FH_ differentiation needs further investigation.

## 6. Future Expectations

Many studies have confirmed that DCs induce the expression of the key transcription factor Bcl6 to further promote T_FH_ cell differentiation through the presentation of antigens in CD4^+^ T cells, the interaction between the costimulatory molecules on the surface of DCs and the corresponding ligands on the surface of T cells, and the secretion of cytokines that function in CD4^+^ T cells. However, many questions remain unanswered. For example, how does the particle size of the antigen influence the duration of action of the peptides/MHCII molecules? Do any other costimulatory factors on DCs influence T_FH_ differentiation in addition to ICOSL and OX40L? Do the cytokines secreted by DCs function in CD4^+^ T cells alone or do they collaborate with other factors to promote T_FH_ differentiation? Lastly, what are the specific mechanisms? Therefore, subsequent in-depth investigations of the influences of the molecules associated with the three types of signals in DCs regarding T_FH_ differentiation and their mechanisms have importance for both the final elucidation of the differentiation and development processes and the understanding of the mechanism of humoral immune responses in the body.
